# Autoantibodies neutralizing type I interferons in patients with life-threatening COVID-19 pneumonia: a meta-analysis from 2020–2026

**DOI:** 10.64898/2026.08.06.26359907

**Published:** 2026-08-10

**Authors:** Elsa Feredj, Qian Zhang, Paul Bastard, Jean-Laurent Casanova, Aurélie Cobat

## Abstract

Autoantibodies neutralizing type I IFNs (AAN–IFN-I) have been found in significant proportions of cases of severe, critical, and fatal COVID-19 pneumonia. We performed a systematic review of 54 studies reporting auto-Abs against type I IFNs and a meta-analysis of 20 studies reporting auto- Abs neutralizing type I IFNs published between 2020 and 2026. The meta-analysis included data for 11,380 SARS-CoV-2-infected individuals from Europe, North America, South America, Asia, the Middle East, North Africa and international multicenter cohorts, including 7,814 with severe or critical disease (69%). The pooled prevalence of AAN–IFN-I was estimated at 7.9% (95% CI, 6.0–10.4). Disease severity was strongly associated with AAN–IFN-I prevalence (OR, 11.7; 95% CI, 7.6–17.9; *P* =5×10⁻²⁹). The pooled prevalence of AAN-IFN-I reached 11.4% (95% CI, 10.2– 12.7%) in patients with severe or critical COVID-19 and 15.3% (95% CI, 12.1–19.2%) in those who died. The prevalence of AAN–IFN-I increased with age in patients with severe, critical, or fatal COVID-19. AAN-IFN-I probably accounted for about 1.1 million of the 7.1 million deaths from COVID-19. AAN-IFN-I are strong, common, global determinants of life-threatening COVID-19 pneumonia.

## Full Text Availability

The license terms selected by the author(s) for this preprint version do not permit archiving in PMC. The full text is available from the preprint server.

